# The Danish Chronic Disease Cohort: using digital footprints to identify chronic disease patterns

**DOI:** 10.1007/s10654-025-01354-2

**Published:** 2026-02-21

**Authors:** Ann Taber, Ricco N. H. Flyckt, Margrethe H. B. Henriksen, Kaire Innos, Wenche Nystad, Lars J. Kjerpeseth, Brit L. Sandgren, Claus Varnum, Malene R. V. Pedersen, Christopher Johansen, Torben F. Hansen, Claus L. Brasen

**Affiliations:** 1 Department of Oncology, Aarhus University Hospital, Aarhus, Denmark; 2 Department of Oncology, Lillebaelt Hospital, Vejle, Denmark; 3 Department of Epidemiology and Biostatistics, National Institute for Health Development, Tallinn, Estonia; 4 Department of Chronic Disease, Norwegian Institute of Public Health, Oslo, Norway; 5 Department of Orthopaedic Surgery, Lillebaelt Hospital, Vejle, Denmark; 6 Department of Regional Health Research, Faculty of Health Sciences, University of Southern Denmark, Odense, Denmark; 7 Department of Radiology, Lillebaelt Hospital, Vejle, Denmark; 8 Department of Biochemistry and Immunology, Lillebaelt Hospital, Vejle, Denmark

**Keywords:** Non-communicable diseases, Cancer, Prevention, National data, Machine learning

## Abstract

Non-communicable diseases (NCDs) represent a significant health burden in Europe, accounting for the vast majority of deaths and consuming a substantial portion of healthcare resources. Despite the largely preventable nature of NCDs through modifiable behavioral risk factors, current prevention efforts remain limited. This project aims to establish a comprehensive data infrastructure to enhance understanding and prevention of NCDs. Leveraging Denmark’s extensive national data resources, this study plans to integrate diverse datasets, including health registers, genomic data, environmental exposures, and national health surveys, covering a total study population up to 8.7 million individuals, including all living and deceased in Denmark since 1975. By integrating these datasets, the project will establish the Danish Chronic Disease Cohort, a platform designed to uncover complex risk patterns associated with non-communicable disease (NCD) development. Future machine learning applications will analyze the range of health determinants, supporting the development of interpretable predictive models to guide targeted prevention and policy initiatives across Europe.

**Supplementary Information:**

The online version contains supplementary material available at 10.1007/s10654-025-01354-2.

## Introduction

Non-communicable diseases (NCDs) which refer to a group of chronic conditions not caused by infectious agents, account for 80% of the disease burden in Europe [1]. The main NCDs include cancer, cardiovascular diseases, chronic respiratory diseases and, osteoarthritis. The term NCD and chronic disease are often used interchangeably, as almost all NCDs are long-term and progressive. Klik eller tryk her for at skrive tekst.. NCDs develop due to a combination of behavioral, environmental, genetic, and physiological factors, with the majority linked to four key behavioral risks: smoking, physical inactivity, alcohol consumption, and unhealthy diets [1, 2]. Tobacco and alcohol use are more prevalent among low socioeconomic groups, who also experience poorer health outcomes, partly due to limited healthcare access [3]. As a result, inequalities in the burden of NCDs persist, both within and between countries, with mental disorders and cancers notably higher in Eastern Europe than in Western Europe [4]. At the same time, most developed countries are experiencing a rapid increase in their aging population, which is expected to further increase the prevalence of NCDs [5, 6]. Aging populations are more likely to have multiple comorbidities, longer durations of chronic disease and accumulated exposure to risk factors [7]. Therefore, now more than ever, a shift toward early prevention is crucial. Prioritizing preventive strategies could lead to improved public health outcomes, lower total healthcare costs, and a reduction in the overall burden of NCDs.

In Denmark, all citizens have equal access to all healthcare, social security services and education due to a tax-fundes public health and educational system. The country also ranks among those with the lowest income inequality, according to the Organisation of Economic Co-operation and Development OECD [8] However, despite equal and free access to public healthcare, disparities persist in disease prevalence and healthcare utilization across the population. Danes with lower educational attainment and income exhibit higher incidence rates of NCDs, such as diabetes, endocrine disorders, respiratory and digestive diseases, skin and musculoskeletal diseases, and oral health problems [9] Trust is a fundamental value in Danish society, extending to healthcare, government, law enforcement, justice, and digital services. As one of the most digitized countries in Europe, Denmark has government institutions dedicated to collecting, storing, and protecting citizens’ personal data. Consequently, the data footprint of each Danish citizen serves as a unique resource for research aimed at improving public health.

In recent years, the exploration of artificial intelligence (AI) in healthcare is rapidly expanding, with a great potential to improve disease predictions and guide targeted interventions in NCD management [10]. AI encompasses both subfields of machine learning (ML) and neural networks to identify novel patterns among large datasets and predict risks related to disease detection, prognosis and treatment response [11, 12]. ML has already demonstrated its effectiveness in predicting a range of NCDs, including breast cancer, diabetes, osteoporosis, and cardiovascular diseases [13–16]. However, the performance and results of ML algorithms are heavily influenced by the quality of the data available for training, which affects factors such as reliability, interpretability, and accountability [17]. Recent studies have emphasized the risks associated with AI in healthcare, which stem from uncertainties in healthcare data, preprocessing differences amongst studies and the non-explainability of ML models, such as complex deep-learning algorithms [18, 19]. It is essential to establish a well-structured framework for data collection and preprocessing, with clear descriptions of all decisions involved in the selection of data sources and datasets prior to analysis. Furthermore, providing a logical, clinically understandable explanation of the ML methods, such asincorporating explainable AI techniques,is also critical for clinicians to interpret model predictions and comprehend related publications.

Since January 2024, the co-funded EU initiative, Joint Action Prevent Non-Communicable Diseases (JA PreventNCD), has focused on addressing the challenges posed by NCDs by supporting strategies and policies aimed at reducing their burden [20]. As part of this initiative, this project was developed with the overall aim of utilizing existing population-based monitoring systems to support data-driven NCD risk prediction, ultimately enhancing future prevention strategies. In this paper, we present the study design and methodology.

## Methods

### Setting and study population

Denmark has a population of more than 5.9 million inhabitants [21]. Since 1968, each resident has been assigned a unique ten-digit personal identification number at birth (CPR, Civil Registration Number). This number allows precise linkage and merging of across Danish national registers, electronic health records (EHR), and socioeconomic and environmental data, among others [22]. The study population comprises all individuals who have resided in Denmark at any point from 1975 up to January 1, 2025, including individuals who have deceased since 1975. In total approximately 8.7 million individuals. The starting year 1975 was chosen as it marks the earliest availability of the registries used to define the project database cohort. This combined with free healthcare for all makes the cohort highly representative for the entire population of Denmark and reduces any bias to an absolute minimum.

### Study design and objectives

**Fig. 1:**
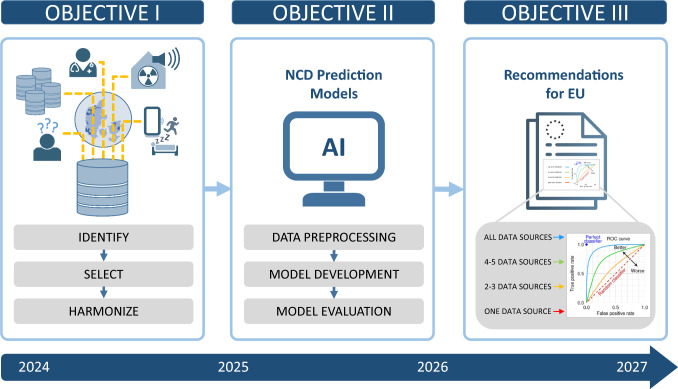
Overview of Study Objectives: The study aimed to: (i) comprehensively align multiple Danish data sources monitoring health determinants, risk factors, and outcomes associated with NCDs; (ii) investigate the use of machine learning to analyze the relationships between risk factors and NCDs for developing prediction models; and (iii) propose improvements in data collection, integration, and analysis to strengthen future NCD prevention strategies

The project was initiated in January 2024 and is structured around three main objectives (Fig. 1). First, to identify, select, and harmonize Danish data sources for monitoring health determinants, risk factors, and outcomes related to NCDs from both clinical and population perspectives. In this context, harmonization refers to the integration of national registries using the unique personal identification number (CPR) to enable linkage at the individual level across multiple data sources and over time. Next, we will analyze the relationships between various risk factors and NCDs, using ML methods to develop robust NCD prediction models. Finally, informed by the findings from the first two objectives, we will provide recommendations to the EU to strengthen future NCD prevention strategies.

### Identification of multiple data sources

**Fig. 2:**
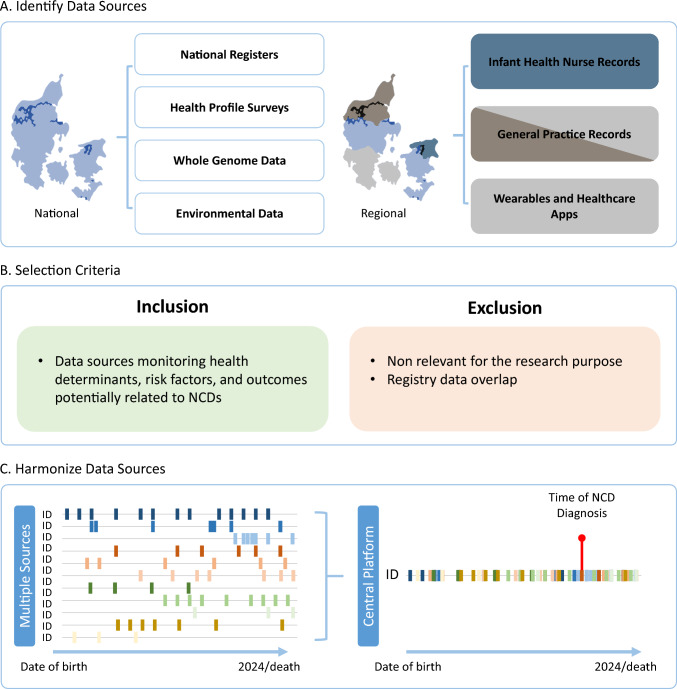
Identification, Selection and Harmonization of Potential Multiple Danish Data Sources. **A** Identification of possible national and regional data sources in Denmark, focusing on sources monitoring health determinants, risk factors, and outcomes related to NCDs. Light gray/dark gray indicate regional data from general practice in both the Northern and Southern Regions of Denmark, while data from wearables and healthcare apps are available only in the Southern Region of Denmark. **B** Inclusion and exclusion criteria for selecting data sources, based on the principle of data minimization. **C** Aggregation of multiple data sources into a central platform, where key health events, such as NCD diagnosis (indicated by a red dot), are highlighted for analysis

The selection process (Fig. 2) follows the principle of data minimization, a core concept in General Data Protection Regulation (GDPR) within the EU. To ensure compliance, variables across data sources were reviewed for overlapping information and relevance, limiting the collection of personal data to only what is directly relevant and necessary to achieve the study’s specified purpose. Authors AT, MBH, RNHF, and CLB conducted the selection process, and all authors reviewed each data source.

All data sources are accompanied by extensive metadata and data dictionaries provided by the registry owners. These include detailed descriptions of all variables, their coding schemes, and, when relevant, the methods used to derive or calculate variables. The quality and completeness of the metadata and data dictionaries vary across registries.

To avoid overlapping data for time-invariant variables (for example, place of birth), we selected one consistent source for each. All time-varying or self-reported variables (for example, smoking status) were included, as we plan to perform cross-source validation, since discrepancies may occur between clinical records and self-reported information. Figure 2

## Data sources

### Danish national registers

Denmark has established a robust data infrastructure that facilitates nationwide register data for both clinical and research purposes. Statistics Denmark manages 468 national registers, encompassing data on education, income, housing, social assistance, and health, some dating back to the 1940s [23]. The Danish Health Data Authority oversees 41 national health registers, which provide comprehensive health data on the entire population, enabling studies of disease development and treatment over time [24]. The Clinical Quality Program’s National Clinical Registries (RKKP) manages 85 quality registers, offering detailed patient-specific data, including diagnostics, treatment, outcomes, and screening patterns [25]. Additionally, the Danish National Archives, The Department of Public Health and the University of Copenhagen maintain 61 conscription registers containing records on Danish men from 1957–1984 and 1987–2020, which include data on education, health, and intelligence [26, 27].

### Danish national health surveys

The National Institute of Public Health has collaborated with The Danish Health Data Authority and performed The Danish National Health Survey in 2010, 2013, 2017, 2021, and 2023. The surveys engaged approximately 700,000 participants who completed a standardized questionnaire, covering sociodemographic factors, quality of life, chronic illnesses, health behaviors, interactions with healthcare services, and social relationships [28]. Additionally, it utilized validated scales such as the 12-Item Short-Form Health Survey (SF-12) v2, the Perceived Stress Scale (PSS), and the CAGE-C test for alcohol abuse screening [29–31]. Each of Denmark’s five regions managed their respective samples, including data collection and reporting, and supplemented the standardized questionnaire with region-specific questions to create unique Regional Health Profiles.

### Danish national genome center

The Danish National Genome Center (DNGC) manages the infrastructure for whole genome sequencing in Denmark, overseeing data storage and analysis within the National Genome Database [31, 32]. All genomic data generated through whole genome sequencing within Denmark’s healthcare system is reported to this central database, enabling the continuous accumulation of comprehensive genetic information. As of April 2025, the database has collected a total of 43,050 genomes.

To ensure data security, all data within the National Genome Database must remain strictly within the DNGC research infrastructure, accessible only through the DNGC Cloud. Currently, it is not possible to gain direct read access to the Danish National Genome Database; however, access options for researchers are expected to be available soon.

### Environmental data sources

#### Noise pollution

The Danish Environment Agency has measured noise pollution along major roads, railways, and within significant urban areas across Denmark. These measurements are taken at locations such as roads or railways. To estimate each individual’s noise exposure, we assess how close their address is to the nearest noise source. By linking addresses to the CPR number, using national registers (like Statistics Denmark), we can indirectly estimate individual exposure levels [33]. According to the World Health Organization (WHO), traffic noise can lead to adverse health effects, such as sleep disturbances, stress, increased blood pressure, heightened risk of cardiovascular diseases, and hormonal impacts [34].

#### Radon exposure

The Danish Health Authority, in collaboration with regional health authorities, has gathered radon exposure data across Denmark, covering more than 3,000 single-family homes distributed across 275 local parishes. Each address can be linked to its corresponding parish through national registers, such as those maintained by Statistics Denmark. The data, aggregated at the municipality level, reflects broader trends rather than specific readings from individual homes [35]. The WHO identifies radon as the second leading cause of lung cancer, responsible for between 3 and 14% of all lung cancer cases [34, 36]. Long-term exposure to elevated radon levels poses significant health risks, particularly in combination with smoking.

#### Air pollution

The European Environment Agency monitors air quality across Europe, including major Danish cities such as Aalborg, Odense, Aarhus, and Copenhagen [37]. The agency tracks key airbone pollutants, such as PM2.5 and PM10, which the WHO has linked to various NCDs, including cardiovascular diseases and cancer [38, 39].

### Danish regional data sources

The Copenhagen Infant Health Nurse Records (CIHNR) cohort includes data on 92,902 infants born between 1959–1967 in Copenhagen, Denmark. It contains information on birth weight, illness, feeding, parental occupation, home hygiene, family structure, child-care, motor development, parent–child relationships, and general health [40]. The cohort enables research on early-life factors linked to later NCDs. A national database containing Infant Health Nurse Records is currently being developed, and we intend to submit a data access application once it becomes available.

Danish general practitioners (GPs) operate under regional contracts while owning their clinics, each managing an average of 1600 patients. Every resident in Denmark has access to a GP, with most services provided free of charge. GPs address most medical cases, with citizens, on average, visiting the primary healthcare system seven times per year [41]. The Center for General Practice at Aalborg University has established a research infrastructure to retrieve, store, and process data from Danish GP electronic patient record (EPR) systems, supporting ongoing research projects in the North Denmark Region [42]. This infrastructure currently includes data from over 80,000 GP patients across Denmark, capturing free-text electronic health records, International Classification of Primary Care, 2nd edition (ICPC-2) codes for diagnoses and symptoms, and discharge summaries from other specialists and private hospitals. This comprehensive dataset provides valuable insights into primary care and can be linked to the progression of NCDs over time. Plans are underway to establish a collaboration between the Region of Southern Denmark and the Center for General Practice at Aalborg University, further enhancing research capabilities and data integration.

Finally, the Region of Southern Denmark has developed the DataDonor app, which enables citizens to directly share health data, including information on exercise and sleep patterns [43]. This real-time data capture through wearable devices (e.g. smartwatches, fitness tracker etc.) presents a promising approach to obtaining up-to-date health information on physical activity.

### NCD or chronic disease definition

For consistency across studies, NCDs will be defined as chronic, non-infectious conditions in line with the WHO definition of NCDs [44]. In projects focusing on cancer, the relevant NCDs will be defined using ICD-10 codes from the Danish Cancer Register and the National Patient Register. For other NCDs that are primarily diagnosed and managed in general practice—such as hypertension, diabetes, chronic obstructive pulmonary disease (COPD), or arthritis—mild and moderate cases may not be captured at hospital level and therefore lack ICD-10 coding. To ensure comprehensive identification of these conditions, we will use a combination of ICD-10 diagnosis codes and Anatomical Therapeutic Chemical (ATC) classification codes, where relevant medications (e.g., antihypertensives, antidiabetics, inhaled bronchodilators) can serve as proxies for disease status. Although Denmark has detailed clinical quality registers for several chronic diseases or specific chronic disease consultations in general practice, the project also aims to apply internationally recognized coding systems such as ICD and ATC wherever possible. This approach will facilitate comparability and data harmonization with international collaborators and future external applications. The final operational definitions for each NCD will be determined within each subproject in collaboration with clinical experts in the relevant field.

Data Infrastructure In this study, data will be collected from multiple sources and securely stored within a central research platform hosted on Statistics Denmark’s servers, a national research platform service offered to Danish researchers. Statistics Denmark operates under strict governance and complies fully with GDPR and ensures a high level of information security through ISO/IEC 27001:2022 certification and continuous external auditing (ISAE 3000) [45]. All data handling follows national confidentiality and access policies.. Additionally, Statistics Denmark’s offers cloud-based services for data preprocessing and analysis, ensuring a streamlined and secure workflow for managing and analyzing the collected data. Analyses will be conducted using Python version 3.7.4 within isolated virtual environments, which enhance reproducibility and modularity in the analysis pipeline.

The central research platform acts as a core infrastructure, supporting the development of various subprojects. Projects will start with focusing on specific NCD, currently lung, breast and colon cancer, cardiovascular disease, chronic obstructive pulmonary disease (COPD), and osteoarthritis are included. Highly specific data, referring to information that is relevant only to a particular subproject and not applicable across others, will not be stored on the central platform but instead accessed and managed within that subproject alone. In the coming years, additional subprojects will be created focusing on other NCDs, to expand NCD research coverage in other specialties research scope, and impact.

### Machine learning approaches

**Fig. 3:**
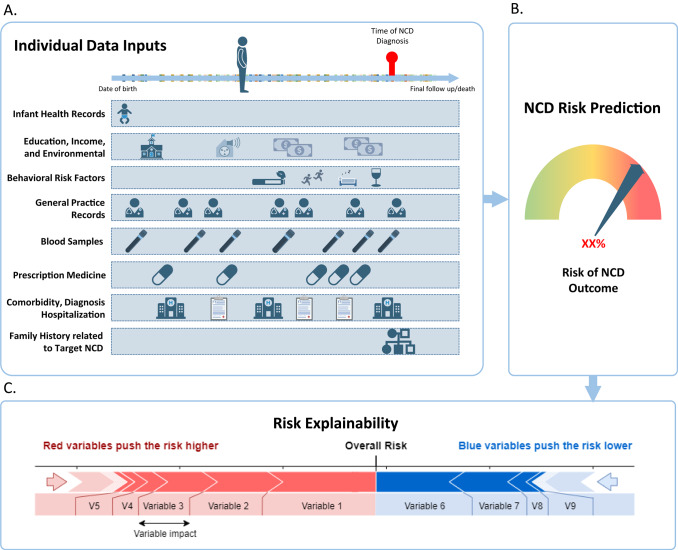
Data Footprint of Individuals Prior to Diagnosis of Specific NCD. **A** Illustration of the types of data collected throughout an individual’s life before the diagnosis of a specific NCD. The timeline progresses from birth (left) to diagnosis and beyond (right); depicting various data points relevant to understanding health trajectories. **B** Example of an NCD Risk Prediction Output for an individual based on their lifetime data inputs. **C** Risk explainability for an individual’s NCD risk as predicted by the ML model. Red bars indicate variables that increase the overall risk score, while blue bars represent variables that decrease it. The length of each bar reflects the impact of each variable on the overall risk, with the overall risk score indicating the individual’s risk of developing the given NCD. Created in BioRender.com

The established central database will contribute to the development of predictive ML models (Fig. 3A-B). Using ML enables a more holistic assessment of NCD risk by analyzing an individual’s entire health history, rather than very limited isolated variables, as in traditional screening [11]. Numerous reporting guidelines have been established to standardize AI-related research across preclinical, translational, and clinical settings [46, 47]. These frameworks provide a structured approach for assessing quality throughout the entire AI prediction model cycle,from development to evaluation and implementation. In this project, these guidelines will form the foundation for developing robust ML models and will help ensure the future safe, effective, and ethical translation of developed prediction models into public health practice. In short, we will follow three main steps:

**Data preprocessing**: This initial step transforms raw data into a format suitable for ML training and evaluation [48]. Key tasks include exploratory data analysis, validating data quality, assessing potential biases, addressing imbalances in outcome variables, handling missing values, normalizing the data, creating new variables through feature engineering and variable selection.

**Model development**: This phase involves experimenting with various ML algorithms to identify the most effective model for the desired prediction. Models of differing complexity, such as logistic regression and neural networks, are developed to balance performance with simplicity. Hyperparameter tuning is conducted to further optimize model performance [49].

**Model evaluation**: The final step assesses the model’s performance and reliability. Evaluation is conducted on a separate validation dataset, using cross-validation and performance metrics aligned with the objective. This step is crucial to ensure that the model has captured genuine patterns rather than overfitting to the training data by detecting correlations in the training dataset that may not generalize to new, unseen data. However, the ability of ML models to capture complex patterns and associations often comes with a trade-off: they are frequently perceived as ‘black boxes,’ making their predictions difficult to interpret. To address this, our study will incorporate Explainable AI techniques [50]. The Shapley Additive Explanations (SHAP) force plot provides a breakdown of the variables influencing a model’s prediction for a given individual, enabling interpretability and enhancing transparency and trust in model-driven insights (Fig. 3C).

### Future perspectives

The Danish Chronic Disease Cohort presents a unique opportunity to leverage a wide range of national data sources for NCD prevention. Collecting data on common risk factors such as smoking, physical activity and harmonizing the data across multiple data sources makes it possible to expand the research topics to include a wide range of NCD’s but also to look into other risk factors, when adjusting for the known risk factors in a large population. This could certainly be all types of cancer, cardiovascular diseases, diabetes, pulmonary diseases, certain eye diseases, fertility, children diseases, birth defects, pregnancy health, early death and many more. We will also be working on a federated learning-approach where possible and where it is needed to collaborate with other research groups in other countries due to GDPR.

Several other European countries have developed similarly comprehensive and interoperable data infrastructures, highlighting the broader scalability and adaptability of this approach across Europe. Estonia and Norway, both collaborators within the JA PreventNCD framework, illustrate in the following sections that well-established national data systems for population-level health monitoring are already in place in other European countries. These parallel systems highlight the potential for cross-country harmonization of data and sharing of analytical methods. Moving forward, this collaboration also provides an important opportunity to externally validate predictive ML models developed within the Danish cohort.

Estonia serves as a strong example of how structured digital systems can support large-scale health research and preventive care. With a population of 1.3 million, Estonia has assigned all residents a unique 11-digit personal identification number since 1992, which enables linkage across various national databases. The central population database is the Estonian Population Registry managed by the Ministry of the Interior. Statistics Estonia manages census data linked with registries holding data on employment, income, education, etc. Population-based nationwide health registries are maintained mainly by National Institute for Health Development.

Klik eller tryk her for at skrive tekst.Klik eller tryk her for at skrive tekst.Klik eller tryk her for at skrive tekst.Klik eller tryk her for at skrive tekst.In Norway, the NCDNOR project has demonstrated the potential to generate new knowledge for the prevention of NCDs by combining data from national registries with information from population-based health studies [51]. As in Estonia and Denmark, each Norwegian resident has a unique personal identifier, enabling longitudinal follow-up and integration across datasets without duplication. Using national mandatory health and administrative registries, Norway combines individual-level data from a wide range of sources (Supplementary Fig. 1). Further details regarding the data sources and infrastructures of Estonia and Norway are provided in Supplementary File 1.

## Discussion

The Danish Chronic Disease Cohort plans to integrate data from multiple national sources into a comprehensive, population-based framework, covering a total study population of up to 8.7 million individuals. The use of unique personal identifiers enables linkage across registries, surveys, and clinical sources. This structure makes the cohort well suited for studying relationships between health determinants, behaviors, and outcomes over time, and for analyzing early risk factors and long-term disease patterns relevant to understanding NCDs. The resource also provides a foundation for developing predictive ML models that could guide future NCD prevention and public health planning. As part of the JA PreventNCD framework, the cohort will further contribute to cross-country collaboration and enable external validation of predictive models across comparable European data infrastructures.

The ability to link data over time is particularly valuable for identifying early risk factors, as seen in prior studies connecting infant weight gain to coronary heart disease in adulthood [52]. Moreover, previous research has demonstrated that EHR) data can enhance clinical predictions by uncovering hidden patterns, such as disease progression and shifts in key variables [53]. This dynamic tracking of changes further strengthens our ability to identify early indicators and improve NCD risk prediction. Comparable comprehensive databases in Denmark include iPSYCH, which focuses on mental health, and the Danish National Birth Cohort (DNBC), designed to investigate causal links between early-life exposures and later disease, with long-term follow-up of over 100,000 pregnant women and their children [54, 55]. However, to our knowledge, no existing database in Denmark integrates such a diverse range of data sources and covers all individuals registered in Denmark (both living and deceased) from 1975 to 2025, corresponding to approximately 8.7 million individual, with the specific aim of predicting multiple NCDs, as proposed for the Danish Chronic Disease Cohort.

While the Danish Chronic Disease Cohort is expected to offer extensive coverage, it is important to recognize that certain data sources only cover specific subpopulations. This could introduce gaps in representation and overlook risk factors in underrepresented groups, potentially affecting the broader applicability of the study’s insights. However, we anticipate that the large size of the Danish Chronic Disease Cohort will help combat selection bias by enhancing diversity, as it encompasses comprehensive data from the entire Danish population. Additionally, while the study adheres to the principle of data minimization to comply with ethical standards for data privacy, the exclusion of certain registries and variables deemed irrelevant may result in missed associations with NCDs. Given the complexity of NCD etiology, even seemingly unrelated factors could play a role in disease development.

In addition to these coverage-related limitations, other potential weaknesses concern data accuracy and consistency across data sources. Misclassification of diseases and variability in disease detection, often influenced by health inequality across vulnerable populations and regions, can affect data accuracy. In addition, differences in disease progression and severity may lead to varying detection rates. External factors such as changes in clinical guidelines, reimbursement schemes, coding practices, healthcare accessibility, treatment-seeking behavior, and pharmaceutical marketing can further influence disease registration. These factors highlight the importance of combining data from multiple registries to more accurately estimate the prevalence and incidence of certain NCDs. Overall, the Danish Chronic Disease Cohort serves as an umbrella framework from which multiple substudies will emerge, with forthcoming publications providing detailed descriptions of the cohort, coverage, potential biases, and methodological considerations.

The second objective of the study,leveraging ML models for NCD prediction, further amplifies its potential, as ML has shown success in detecting complex patterns within high-dimensional data that traditional methods often overlook [11]. However, the effectiveness of ML models depends heavily on the quality of data used for training, and variability in data quality across sources can affect the reliability of ML-driven insights [7]. Issues such as missing data, incomplete records, and biases in observational studies have been shown to compromise the accuracy of predictive models [17]. Addressing these data quality concerns will be crucial to ensuring the robustness and reliability of our ML-based predictions.

The use of ML in healthcare also presents ethical challenges, particularly with the non-explainability of many machine learning algorithms, especially complex deep learning models, which often function as mentioned ‘black boxes’. This lack of transparency can make it difficult for clinicians to trust and understand how predictions are generated. To address these concerns, we will prioritize the use of explainable ML models and implement validation processes in future studies to ensure that our ML-driven insights are transparent, reliable and aligned with the EU AI Act [56] to the fullest extent possible. Moreover, data drift represents another important challenge when developing and maintaining predictive models over time. National-level changes such as economic recessions may change the relationships between predictors (e.g. yearly income) and outcomes (e.g. cancer). To combat this, continuous monitoring of the predictive models and retraining when new data becomes available is essential, to ensure the models remain accurate and relevant.

## Conclusion

This project presents an ambitious approach to reducing the burden of NCDs through data-driven prevention strategies. The strengths of the project is based on the comprehensive use of Denmark’s extensive data resources, enabling a detailed understanding of health determinants across the life course. By harmonizing data from multiple sources and utilizing ML for predictive insights, our project is well-positioned to identify early risk factors, which may enhance NCD prevention efforts. However, challenges such as data variability, subpopulation representation, and the ethical implications of using complex ML models must be carefully addressed to ensure the application of future findings.

## Supplementary Information

Below is the link to the electronic supplementary material.

Supplementary file1 (DOCX 103 KB)
