# Supplementary material for: The Danish Chronic Disease Cohort: using digital footprints to identify chronic disease patterns
Source: Eur J Epidemiol. 2026 Feb 21;41(3):377–87. doi: 10.1007/s10654-025-01354-2 (PMC13222317; doi:10.1007/s10654-025-01354-2)
Supplement: Supplementary file 1 — Supplementary file1 (DOCX 103 KB) [file 10654_2025_1354_MOESM1_ESM.docx]

## **Supplementary File 1**

**Estonia**

Estonia maintains several high-quality, population-based health registries, including the Estonian Cancer Registry (1) that has data since 1968, Medical Birth Registry, Causes of Death Registry, Cancer Screening Registry, etc. Estonian Health Information System created in 2008, is a central national database than enables health care service providers to exchange data (2) . Data insertion to the system is obligatory for health care providers and data are also visible to the patient via the Health Portal. To enable development of personalized medicine services and to improve preventive care and treatment plans based on individual genetic profiles, a national IT infrastructure was created in 2019-2023 including consent management system, genetic data information system, monitoring and management platform for genetic risk models, computation environment for personalized medicine, and personalized health and treatment recommendation system (3). Since 2023, the infrastructure is managed by Estonian Health Insurance Fund. Estonian Biobank has established a population-based biobank with a current cohort of over 200 000 individuals (20% of adult population) (4). The country's primary data exchange platform, X-Road, facilitates the communication of different information systems across the governmental sector.

**Norway**


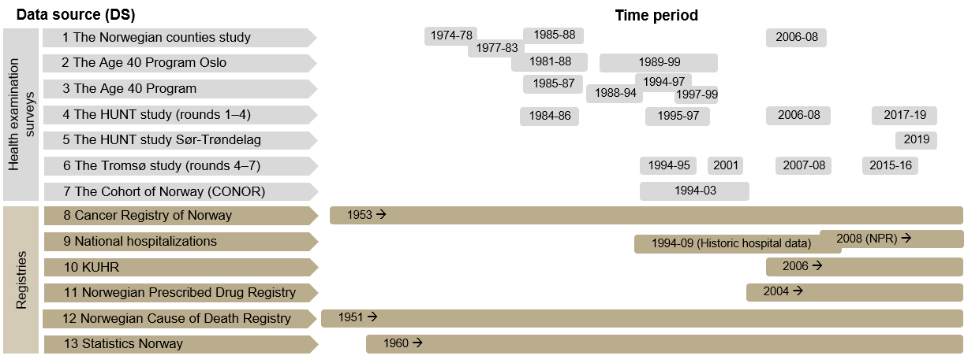


**Supplementary Figure 1: Overview of Norwegian data sources**. HUNT: Trøndelag Health Study; NPR, Norwegian Patient Registry; KUHR, Norwegian Control and Payment of Health Reimbursements Database. CONOR is a collection of data from Tromsø4-Tromsø5; HUNT2; ‘HUSK’ (The Hordaland Health Study); ‘Oslo II’ (The second Oslo Study); ‘HUBRO’ (The Oslo Health Study); ‘I-HUBRO’ (The Oslo Immigrant Health Study); ‘OPPHED’ (The Oppland and Hedmark Health Study); ‘TROFINN’ (The Troms and Finnmark Health Study), and; ‘MoRo II’ (The second part of the Romsås in Motion Study).

Overview of Norwegian data sources are illustrated in supplementary figure 1. Data sources (DS) 1-7 provide information on behavioural and biological NCD risk factors in a sub sample of about ~800,000 individuals (~1.2 million person-observations) (5). The national mandatory health registries (DS 8-12) cover the entire population: DS 8 includes data on nearly all cancer cases; DS 9 and 10 includes data from specialist and primary healthcare, respectively; DS 11 contains information on all prescriptions dispensed in community pharmacies; and DS 12 includes causes of death at ~98% completeness. Finally, DS 13 compiles sociodemographic and family data for all Norwegian residents since 1960 from the National Registry, censuses, education databases, tax administration, and welfare administration (6).

# **Supplementary Reference**

1. Rahu M, Rahu K. The Estonian Cancer Registry: foundation and further history. Acta Oncol (Madr) [Internet]. 2018 Oct 3 [cited 2025 Mar 27];57(10):1407–10. Available from: <https://medicaljournalssweden.se/actaoncologica/article/view/24643>
2. Health information system | TEHIK [Internet]. [cited 2025 Mar 27]. Available from: <https://www.tehik.ee/en/health-information-system>
3. Personalised medicine | Tervise Arengu Instituut [Internet]. [cited 2025 Mar 27]. Available from: <https://www.tai.ee/en/personalisedmedicine>
4. Estonian Biobank [Internet]. [cited 2025 Mar 27]. Available from: <https://genomics.ut.ee/en/content/estonian-biobank>
5. Dalene KE, Lergenmuller S, Sund ER, Hopstock LA, Robsahm TE, Nilssen Y, et al. Clustering and trajectories of key noncommunicable disease risk factors in Norway: the NCDNOR project. Scientific Reports 2023 13:1 [Internet]. 2023 Sep 2 [cited 2025 Mar 27];13(1):1–12. Available from: <https://www.nature.com/articles/s41598-023-41660-x>
6. NYSTAD W, HJELLVIK V, LARSEN IK, ROBSAHM TE, SUND ER, KROKSTAD S, et al. National health registries – a ‘goldmine’ for studying non-communicable disease occurrence in Norway – the NCDNOR project. Scand J Public Health [Internet]. 2023 Dec 1 [cited 2025 Mar 27]; Available from: <https://journals.sagepub.com/doi/abs/10.1177/14034948231214580>
